# Variability, shift‐specific workloads and rationed care predictors of work satisfaction among Registered nurses providing acute care: A longitudinal study

**DOI:** 10.1002/nop2.1160

**Published:** 2021-12-15

**Authors:** Mary Abed Al Ahad, Martine Elbejjani, Michael Simon, Dietmar Ausserhofer, Huda Abu‐Saad Huijer, Suzanne R. Dhaini

**Affiliations:** ^1^ School of Geography and Sustainable Development University of St Andrews St Andrews UK; ^2^ Clinical Research Institute Faculty of Medicine American University of Beirut Beirut Lebanon; ^3^ Institute of Nursing Science University of Basel Basel Switzerland; ^4^ Inselspital Bern Switzerland; ^5^ College of Health‐Care Professions Claudiana Bozen Italy; ^6^ Faculty of Health Sciences University of Balamand Balamand Lebanon

**Keywords:** hospital, longitudinal, nursing, patient‐to‐nurse ratio, rationing of care, shift‐work satisfaction, workload

## Abstract

**Aims:**

The aim of this study was to explore nurses’ shift‐work satisfaction variability across time and its shift‐specific predictors: perceived workload, patient‐to‐nurse ratio and rationing of nursing care.

**Design:**

Longitudinal study of 90 Registered nurses (*N* = 1,303 responses) in a Lebanese hospital over 91 days of data collection.

**Methods:**

Intraclass correlation coefficients (ICCs) were computed to determine shift‐work satisfaction variability between individual nurses and working‐unit clusters. Generalized linear mixed models were used to explore the workloads and rationed care predictors of nurses’ shift‐work satisfaction separately for day and night shifts.

**Results:**

Variability in shift‐work satisfaction was noted between individual nurses in day (ICC = 0.43) and night shifts (ICC = 0.37), but not between medical/surgical units. Nurses satisfied with their shift‐specific work were less probably to ration necessary nursing care (OR = 0.68; 95% CI = 0.60–0.77) in day shifts and to perceive high workload demands in both, day (OR = 0.29; 95% CI = 0.23–0.37) and night (OR = 0.29; 95% CI = 0.18–0.47) shifts. Monitoring and lowering workload demands while observing rationing of care is necessary to improve nurses’ shift‐work satisfaction.

## 1 INTRODUCTION

Nurses are a valuable resource in health care (Rivaz et al., 2017). Shortage of nurses is a problem faced worldwide, and it is often attributed to nurses’ poor satisfaction with their overall nursing job and career opportunities, which leads to high attrition rates (Gardulf et al., 2008; Senek et al., 2020). Poor job satisfaction refers to the situation of emotional and physical fatigue accompanied by intrinsic demotivation and an intention of leaving the nursing job (Baljoon et al., 2018; Galletta et al., 2016). Despite the wide literature on understanding the determinants of nurses’ job satisfaction and its associated nursing and patient care outcomes, to the best of our knowledge, there is no published research on nurses’ shift‐work satisfaction. Shift‐work satisfaction refers to nurses’ satisfaction with the quality and quantity of work completed on their respective shifts. Based on the research done by Nadinloyi et al. (2013), nurses’ job satisfaction is mainly linked to two important factors including work environment and quality/quantity of work, which nurses undertake on their respective shifts (Nadinloyi et al., 2013). Therefore, nurses’ shift‐work satisfaction forms an integral part of nurses’ overall job satisfaction, yet the two concepts are distinct and have different research implications. Nurses’ overall job satisfaction is based on whether nurses are satisfied with their nursing job/career (i.e. it has a more cross‐sectional overall focus), while nurses’ shift‐work satisfaction is based on nurses’ satisfaction with the quantity/quality of work they accomplished on their respective shift (i.e. it has a longitudinal more focused dimension). In fact, nurses’ shift‐work satisfaction can reveal the dynamics and implications of each shift enabling more focused shift‐specific strategies rather than general strategies derived from the overall job satisfaction literature.

This study aims to understand the predictors of nurses’ shift‐work satisfaction, which adds novelty to the existing nursing research. Specifically, our study aims to explore whether nurses’ shift‐specific work satisfaction is associated with rationed/missed patient care and workload predictors in acute care setting. This will provide evidence for policies and management strategies tailored to enhance the working conditions for nurses in acute care hospitals, which will improve nurses’ work satisfaction and the quality of patient care in ways that have the most impact.

Given the absence of research on nurses’ shift‐work satisfaction (i.e. whether nurses are satisfied with the quantity/quality of work they have completed on their respective shifts), the background section provides a literature review on the predictors of nurses’ job satisfaction (i.e. satisfaction with the overall nursing job and its working conditions)—a closely related topic—and its associated nursing and patient care outcomes. The following existing empirical review will provide an overview on the impact of the workplace and patient care predictors on nurses’ overall job satisfaction. This will serve as a basis to evaluate by proxy whether these predictors affect in turn the nurses’ shift‐work satisfaction.

## 2 BACKGROUND

Nurses’ poor job satisfaction has been linked to high burnout rates, absenteeism and an intention of leaving the nursing job (Aiken et al., 2012; Gardulf et al., 2008; Nantsupawat et al., 2017). This in turn has demonstrated serious consequences on the quality and safety of patient care, including elevated rates of hospital‐acquired urinary tract and surgical site infections (Cimiotti et al., 2012), in addition to higher mortality and failure to rescue outcomes (Aiken et al., 2003).

Creating a favourable work environment for nurses with low levels of work‐related stress can alleviate the negative impacts of nurses’ poor job satisfaction on patient care quality (Nantsupawat et al., 2017). Based on Herzberg (1966) theory, a favourable work environment is mainly composed of two elements: (1) hygiene which includes good salary and supervision and (2) motivation which refers to recognition and achievement (Herzberg, 1966). In accordance with that, prior research has shown that several factors resulted in nurses’ poor job satisfaction including low wages, scarcity of job benefits, short tenure, high workload demands, inadequate staffing resources, uneven distribution of work tasks, lack of career development opportunities, lack of nurse manager's leadership and support, and absence of teamwork climate and collaboration in the nursing team and with physicians and other healthcare professionals (Chang et al., 2009; Gardulf et al., 2008; Graham et al., 2011; Hayes et al., 2015; Kemper et al., 2008; Khamisa et al., 2015; Senek et al., 2020; Suliman & Aljezawi, 2018). Organizational commitment factors such as professional status, nurse's autonomy recognition, possessing control over practice environment, participating in decision making, ethical frameworks and institutional policies are also predictors of nurses’ job satisfaction (Gardulf et al., 2008; Gillet et al., 2013; Huang et al., 2012; Kalisch et al., 2011).

Poor job satisfaction was also linked to missed nursing care. For example, in her cross‐sectional study, Kalisch et al. (2011) investigated 3,135 Registered nurses (RNs) and 939 assistant nurses, and observed less missed care when nurses reported a higher rate of job satisfaction (Kalisch et al., 2011). While the majority of nurses’ job satisfaction research is concentrated on the socio‐demographic characteristics, work environment and organizational determinants, few studies have considered the link between self‐reported rationing of care—defined as “the withholding or failure to carry out necessary nursing tasks due to inadequate time, staffing level, and/or skill mix” (Schubert et al., 2008)—and nurses’ job satisfaction. Methodologically, existing studies used either a qualitative approach or a cross‐sectional study design to assess this relationship, hence overlooking nurses’ job satisfaction pattern over time. Given the dynamic nature of the healthcare setting and patient care, investigating nurses’ perceptions of work‐related job satisfaction (i.e. satisfaction with the quantity/quality of performed work) at the shift level and its association with rationed care and workloads using a longitudinal design is highly needed.

Accordingly, the overall objective of this paper was to study nurses’ shift‐work satisfaction, which is a shift‐time‐specific outcome. Unlike the common concept of nurses’ job satisfaction, shift‐work satisfaction is more time‐sensitive in longitudinal studies. Specifically, our study aims to examine (1) the variability of nurses’ shift‐work satisfaction—defined as the nurses’ satisfaction with the quantity/quality of work they achieved on their respective shift—across time per type of shift and working unit in a Lebanese acute care hospital and (2) the shift‐specific workloads and rationed care predictors of nurses’ work satisfaction including implicit rationing of care, perceived workload, patient‐to‐nurse ratio and weekend‐shifts.

## 3 STUDY DESIGN

This study is a sub‐study of the “Implicit Rationing of Nursing Care Among Lebanese Patients” (RATIONAL) project. It is a 2‐year funded project (2018–2020) that assesses implicit rationing of nursing care in Lebanese hospitals across time. RATIONAL consists of a cross‐sectional baseline survey (phase 1) followed by a longitudinal observational survey of multiple follow‐up assessments over 91 days (phase 2) for a sample of 317 RNs working on 19 units in two large Lebanese urban hospitals. The project collected data on nurses' rationing of care, hospital administration reported nurse‐sensitive indicators, nurse staffing levels and patient mortality records. Further details on the “RATIONAL” project are described elsewhere (Dhaini et al., 2019). The current paper is a secondary longitudinal data analysis of phase 2 of the project using a sample of 90 RNs working in one Lebanese teaching and tertiary referral hospital over 91 days of data collection.

## 4 METHODS

### 4.1 Sample and setting

The study sample included 90 RNs recruited from one Lebanese teaching and tertiary referral hospital/medical Centre using a convenient sampling approach which considered all the RNs who have been working in the hospital for a minimum of 1 month in direct patient care and who do a minimum of 8 hr/week on five medical and three surgical acute care units.

The hospital setting includes 304 beds and allows for an 8‐hr shift system of day, evening and night as well as a 12 hr of day and night shift system. The hospital provides patient care across different departments including geriatrics, oncology, psychiatry, medical, surgical, obstetrics, paediatrics, critical care, emergency and surgical services.

### 4.2 Data sources

Data used for this study were captured using three questionnaires: (1) nurse personnel questionnaire that captured nurses’ shift‐work satisfaction, implicit rationing of care and perceived workload; (2) nurse managers’ questionnaire which was used to get the staffing level on a shift basis; and (3) nursing administration questionnaire which included questions on the socio‐demographic variables (gender, age groupings and years of nursing experience) of the recruited RNs. It should be noted that socio‐demographic data were collected in an aggregated manner for anonymity, as recommended by the ethics committee of the project institution, from a total of 102 RNs divided as 90 RNs working in five medical and three surgical units and 12 RNs working in two paediatric acute care units. Therefore, this study includes only the 90 RNs working in five medical and three surgical units, while the 12 RNs working in the two paediatric units were excluded due the unique patient care needs of that unit.

### 4.3 Data collection

Between 1 September and 31 December 2018, 90 RNs were recruited and filled daily the nurse personnel questionnaire, over a period of 91 consecutive days (T1–T91). The data collection phase was divided into two phases based on the type of the worked shifts. Phase one data (September 1st–October 31st, equivalent to 61 days: T1–T61) were filled by RNs working on day shifts only, and phase two data (1 November–30 November equivalent to 30 days: T62–T91) were filled by RNs working on night shifts only. A low number of RNs (*n* = 5) worked in evening shifts, and thus, their responses were collected in phase one and merged with the day shift responses.

Data collection was performed using a paper‐based version of the nurse personnel questionnaire distributed through the nursing administration to the nurse managers of each of the five medical and three surgical acute care units at the start of the data collection period (on 1 September 2018). The nurse managers in turn ensured the distribution of these paper‐based questionnaires daily over a period of 91 days to the RNs working in their respective units as described above. Each RN was instructed to complete one copy of the questionnaire on his/her own following each worked shift and without input from other nurses who had worked the same shift. Completed surveys were placed in a sealed envelope to ensure confidentiality. At the end of the data collection period (on 31 December 2018), survey envelops were collected from the nursing administration office by a member from the research team. Responses were digitized using Excel software. Surveys were then placed in a cabinet under double lock.

### 4.4 Study variables

This study included the nurses’ shift‐work satisfaction outcome and a list of six independent variables that were selected a priori based on the predictors identified in the literature review. The outcome and all the six independent variables were measured repeatedly across time (T1–T61 for day shifts and T62–T91 for night shifts).

*Nurses’ shift‐work satisfaction* was measured as a binary variable by asking the RNs if they were satisfied with the quality and quantity of work they achieved in their current shift (no = 0, yes = 1).

*Implicit rationing of nursing care* independent variable was assessed using 14 nursing activities from the five sub‐scales of the “Basel extent of rationing of nursing care (BERNCA)” instrument. The BERNCA is internally consistent and homogenous; the stated inter‐item correlation mean of the scale is 0.39, which signifies a decent consistency with a Cronbach’s alpha of 0.93 (Schubert et al., 2007). Nurses were requested to choose the tasks that they rationed on their respective shift (0 = activity performed; 1 = activity rationed). The 14 included nursing activities involved *Providing* “daily living support”: (1) “partial/sponge bath”; (2) “skin care”; (3) “oral care”; “Documentation”: (4) “patient care plans”; (5) “documenting/evaluating care”; “Rehabilitation, Instruction and Education”: (6) “emotional/psychosocial support”; (7) “training/educating patients”; “Monitoring/safety”: (8) “positioning patients”; and “Caring and Support”: (9) “preparing patients for tests/therapies”; (10) “assessment for newly admitted patients”; (11) “supporting patients with food/oral intake”; (12) “preparing patients for hospital discharge”; (13) “attending to patients who had rung promptly in <5 min”; and (14) “monitoring patients”. The 14 rationing of care questions were summed up for each response to generate one rationing of care score that ranges from 0 (none of the 14 activities is rationed) to 14 (all the 14 activities are rationed).

*Perceived workload* independent variable was measured using the “NASA Task Load Index (NASA‐TLX)” six‐item scale (Hoonakker et al., 2011). This scale has a Cronbach’s alpha of 0.72 (Hart & Staveland, 1988). The “NASA‐TLX” six‐item scale evaluates how much mental (e.g. thinking, deciding, calculating, remembering, looking and searching), physical (e.g. pushing, pulling, turning, controlling and activating), temporal (e.g. time pressure due to the task load), frustration (e.g. feeling insecure, discouraged, irritated, stressed and annoyed), effort (e.g. mental and physical effort to accomplish the required tasks) and performance (job satisfaction performance) demands are needed to perform the required tasks at the workplace on a scale of 0 to 10 (Dhaini et al., 2020). Based on the approach of similar research (Bustamante & Spain, 2008; Hoonakker et al., 2011; Soria‐Oliver et al., 2018), the unweighted average of the six items of the NASA‐TLX scale was computed to generate an overall workload score for each response.

*Patient‐to‐nurse ratio* was used as an assessment of the Staffing levels. The ratio was computed by dividing the number of patients by the number of RNs in each shift and unit based on the data supplied by the nurse managers.

*Time trend* represents the continuous data collection time over 61 days (*T1–T61*) in the day shifts and over 30 days (*T62–T91*) in the night shifts.

*The weekend effect* was captured as a binary variable (0 = weekday; 1 = weekend/national holiday), and the *type of work unit* was coded as 1 = medical unit and 2 = surgical unit.

### 4.5 Ethical approval

The Ethical Institutional Review Board granted permission to conduct this research project on 1 June 2018 (SBS.2017‐0418). The participating hospital informed the research team in writing its willingness to participate. Filling the surveys by RNs was considered as an informed consent.

### 4.6 Data analysis

We performed a separate data analysis for each of the day and night shift responses because our study involved 61 measurement days (T1–T61) for the day shift versus 30 measurement days (T62–T91) for the night shift. Intraclass correlation coefficients (ICCs) were computed to assess the potential basis of clustering (i.e. between‐group variability: *between individual RNs* and between *the surgical and medical units*) for the shift‐work satisfaction outcome in the day and night shifts. An ICC >0.30 indicated the presence of moderate to high variability (Lajos et al., 2014) between the responses received from the different clusters of individual RNs and units. Given the study design (repeated longitudinal assessments of nurses’ shift‐work satisfaction across time) and the between‐group variability in individual RNs clusters (ICC of 0.43 in the day shift and 0.37 in the night shift), the percentage of nurses’ shift‐work satisfaction outcome and the mean of each of the rationing of care, workload and patient‐to‐nurse ratio work environment factors was calculated using mean response predictions from generalized linear mixed (GLMM) models in both the day and night shifts. Binomial family and logit link were specified in the GLMM models, accounting for the between nurses response variability as random effects. GLMM models were also used to determine the association of the six independent variables with the shift‐work satisfaction outcome over 61 days in the day shift and 30 days in the night shift, accounting for the clustering in the repeated shift‐work satisfaction responses among individual RNs in random effects. The independent variables including rationing of nursing care, perceived workload, patient‐to‐nurse ratio, weekend effect, type of unit and time trend were entered simultaneously as fixed effects into the GLMM models. In a sensitivity analysis and to check which of the 14 rationing of care tasks were affecting nurses’ shift‐work satisfaction, we repeated the same models replacing the rationing of care independent variable measured as the number of rationed nursing activities for each response with the 14 tasks. Separate GLMM models were computed for each of the 14 rationing of care tasks to avoid collinearity problems. Odd ratios (ORs) and 95% confidence intervals (CI) were reported. All data analyses were conducted in STATA (StataCorp. 2015. Stata Statistical Software: Release 14: StataCorp LP), and statistical significance was considered at *p*‐value <.05.

## 5 RESULTS

### 5.1 Description of the nurses’ responses and socio‐demographics

**FIGURE 1:**
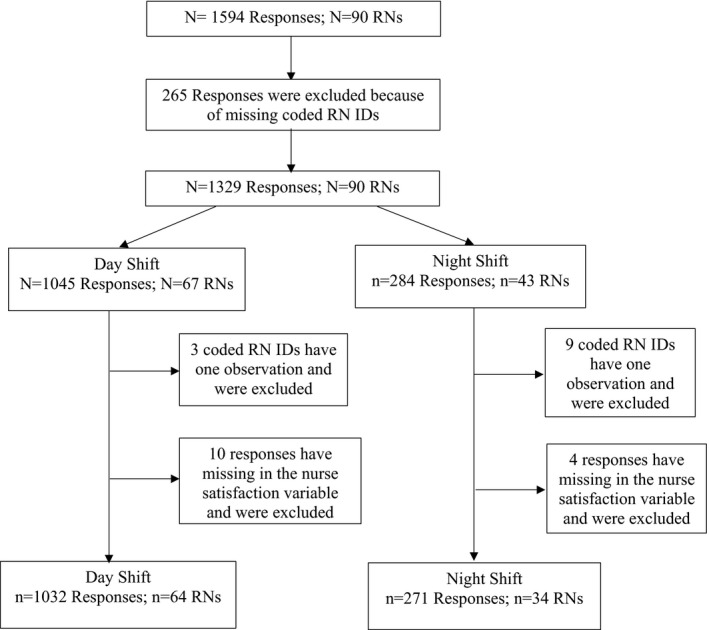
Descrption of the longitudinal survey responses

We received a total of 1,594 responses from the 90 recruited RNs. Individual RN identification codes (IDs) were absent from 265 questionnaires. Some IDs had only one observation during the day or night shifts and some responses had missing values in the shift‐work satisfaction question (Figure 1). Therefore, all the results in the upcoming sections of the paper will be based on a total of 1,303 surveys from 90 RNs, distributed as 1,032 responses from 64 RNs who worked day shifts and 271 responses from 34 RNs who worked night shifts. Out of the 90 RNs, there was an overlap of 8 RNs who worked in both the day and night shifts (Figure 1).

Table 1 provides a summary of the socio‐demographics collected in an aggregated format for the 90 RNs working on the medical and surgical units in addition to the 12 RNs working in the paediatric units (*N* = 102). Most RNs (*n* = 90, 88%) were females and had 2–5 years of nursing experience (*n* = 37, 66%). Around half of RNs (*n* = 48, 47%) aged between 20 and 25 years, and more than a third (*n* = 36, 35%) were in their 26th to 30th of age (Table 1).

**TABLE 1:** Socio‐demographic characteristics of the RNs working in the medical, surgical and paediatric acute care units of the recruited hospital (*N* = 102 RNs)

Socio‐demographics		*N* (%)
Gender	Female	90 (88)
Age (years)	20–25	48 (47)
	26–30	36 (35)
	31–35	3 (3)
	36–40	3 (3)
	41–45	4 (4)
	46–50	4 (4)
	>50	4 (4)
Nursing experience (years)	<2	26 (25)
	2–5	37 (36)
	6–10	24 (24)
	11–15	2 (2)
	16–20	4 (4)
	>20	9 (9)

### 5.2 Description of the nurses’ shift‐work satisfaction and its variability

The analysis of the ICCs indicated the presence of variability in the shift‐work satisfaction responses between individual RNs in both the day (ICC = 0.43) and night (ICC = 0.37) shifts. On the contrary, negligible variability was detected between the clusters of medical and surgical units (Table 2). Hence, accounting for the variability between the RN‐cluster responses, most of the RNs were satisfied with the work they achieved during both, the day (67.89%) and night (84.96%) shifts (Table 2).

**TABLE 2:** Description of the nurses’ shift‐work satisfaction outcome during the day shift (*n* = 1,032, *n* = 64 RNs) and the night shift (*n* = 271, *n* = 34 RNs)

Type of shift	Outcome	%	Intraclass correlation coefficient (ICC)	
			RN ID^a^ (95% CI)	Working unit^b^ (95% CI)
Day shift	Nurse shift‐work satisfaction^c^	67.89	0.43 (0.31, 0.57)	0.002 (0.00, 0.22)
Night shift	Nurse shift‐work satisfaction^c^	84.96	0.37 (0.18, 0.61)	0.006 (0.00, 0.89)

Note

ICC ≥0.30 indicates the presence of homogeneity in clusters of individual RN responses and working units.

^a^ RN ID, ID for each individual RN.

^b^ Working unit: 1 = medical unit, 2 = surgical unit.

^c^ Nurse shift‐work satisfaction: 0 = no, 1 = yes.

### 5.3 Description of the work environment independent variables

Accounting for the individual RNs repeated data, nurses rationed on average 2 out of the 14 necessary nursing tasks in the day shift and 3 out of the 14 necessary nursing tasks in the night shift (Table 3). High workload demand was observed in the sample, with a mean score of 6.75 (scale range 0 to 10) in the day shift and a mean score of 5.89 (scale range 0 to 10) in the night shift. Patient‐to‐nurse ratio was higher in the night shift (mean score = 9.54) as compared to the day shift (mean score = 5.90) (Table 3).

**TABLE 3:** Description of the work environment factors during the day shift (*n* = 1,032, *n* = 64 RNs) and the night shift (*n* = 271, *n* = 34 RNs)

	Mean	Mean
	Day shift	Night shift
Rationing of care^a^	2.10	3.40
Workload^b^	6.75	5.89
Patient‐to‐nurse ratio^c^	5.90	9.54

^a^ Rationing of care: number of rationed nursing activities out of the 14 necessary nursing activities per one response.

^b^ Workload: unweighted mean score of six scale items (mental, physical, temporal, frustration, effort and performance demands).

^c^ Patient‐to‐nurse ratio: number of patients÷number of Registered nurses.

### 5.4 Predictors of the nurses’ shift‐work satisfaction

Analysis showed the presence of a negative association between nurses’ shift‐work satisfaction and the number of rationed nursing tasks (OR = 0.68, 95% CI = 0.60–0.77), only in day shifts but not in night shifts (Table 4). Sensitivity analysis revealed statistically significant negative associations between nurses’ shift‐work satisfaction and rationing of each of the following nursing tasks during day shifts: partial/sponge bath care (OR = 0.47, 95% CI = 0.24–0.93), setting patient care plans (OR = 0.44, 95% CI = 0.27–0.74), documenting/evaluating care (OR = 0.27, 95% CI = 0.14–0.53), emotional/psychological support (OR = 0.26, 95% CI = 0.15–0.46), positioning patients (OR = 0.42, 95% CI = 0.23–0.78), preparing patients for tests/therapies (OR = 0.39, 95% CI = 0.17–0.93), supporting patients with food/oral intake (OR = 0.46, 95% CI = 0.25–0.86) and monitoring patients as necessary (OR = 0.48, 95% CI = 0.27–0.86) (Appendix: Table A1).

**TABLE 4:** Generalized linear mixed models of nurses’ shift‐work satisfaction in the day shift (*n* = 1,032 responses, *n* = 64 RNs) and in the night shift (*n* = 271, *n* = 34 RNs)

	Nurse satisfaction in the day shift	Nurse satisfaction in the night shift
	OR (95% CI)	OR (95% CI)
Time trend^a^	0.98 (0.97, 0.99)*	0.97 (0.91, 1.03)
Rationing of care^b^	0.68 (0.60, 0.77)**	1.10 (0.91, 1.33)
Weekend/holiday^c^	1.12 (0.71, 1.75)	0.69 (0.23, 2.11)
Workload^d^	0.29 (0.23, 0.37)**	0.29 (0.18, 0.47)**
Working unit^e^	0.42 (0.11, 1.62)	0.23 (0.04, 1.40)
Patient‐to‐nurse ratio^f^	0.90 (0.65, 1.23)	0.70 (0.43, 1.13)

^a^ Time trend: data collection time over 61 continuous days (T1–T61) for the day shifts and over 30 continuous days (T62–T91) for the night shifts.

^b^ Rationing of care: number of rationed nursing activities out of the 14 necessary nursing activities per one response.

^c^ Weekend/holiday: 0 = weekday, 1 = weekend or holiday.

^d^ Workload: unweighted mean score of six scale items (mental, physical, temporal, frustration, effort and performance demands).

^e^ Working unit: 1 = Medical unit, 2 = surgical unit.

^f^ Patient‐to‐nurse ratio: number of patients÷number of Registered nurses per unit per shift.

* *p*‐value < .05

** *p*‐value < .01.

Nurses’ shift‐work satisfaction was negatively associated with perceived workload in both, day (OR = 0.29, 95% CI = 0.23–0.37) and night (OR = 0.29, 95% CI = 0.18–0.47) shifts. No statistically significant association was found between shift‐work satisfaction and patient‐to‐nurse ratio in both types of shifts (Table 4).

The type of working unit and working on a weekend versus weekday shifts was not related to nurses’ reported shift‐work satisfaction. Finally, nurses working during the day shift tended to report lower satisfaction with the work they achieved on their respective shifts across time (OR = 0.98, 95% CI = 0.97–0.99) (Table 4).

## 6 DISCUSSION

This study examined the variability of nurses’ shift‐work satisfaction across time per shift, in addition to the effect of implicit rationing of care and workload factors (perceived workload and patient‐to‐nurse ratio) on nurses’ shift‐work satisfaction. Importantly, our study showed that nurses who are less satisfied with their work are more probably to self‐report higher demands of workload and to ration higher numbers of necessary nursing tasks on their respective shifts, particularly for nurses working day shifts. Additionally, no statistically significant associations were noted between nurses’ shift‐work satisfaction and the objective workload measure of patient‐to‐nurse ratio.

Descriptive analysis revealed that the majority of RNs were satisfied with their work on respective shifts. Particularly, those working in the night shift reported higher rates of satisfaction compared with those working day shifts. This could potentially be explained by the nature and dynamics of day shifts characterized by higher patient demands and nursing tasks. For instance, diagnostic and therapy hospital‐related processes are mainly performed on patients between 8:00 a.m. and 5:00 p.m. In addition, elective surgeries and hospital admissions are rarely scheduled during the night, except for emergency cases, a practice that occurs in many major hospitals (Debergh et al., 2012). This reflects less workload demands, and hence more satisfaction among nurses working in the night shift versus the day shift.

Analysis of the ICC revealed high variability between the individual nurse responses, and negligent variation between the medical and surgical acute care units. Our results indicate that nurses’ shift‐work satisfaction is mainly influenced by subjective factors (e.g. perceived workload) that are unique for each nurse rather than unit‐specific objective measures (e.g. specific working tasks and responsibilities or patient‐to‐nurse ratio). It is worth noting that a negative association between nurses’ shift‐work satisfaction and perceived workload in both day and night shifts was found, while patient‐to‐nurse ratio—an objective measure of the workload—was not related to nurses’ shift‐work satisfaction. The negative correlation between nurses’ shift‐work satisfaction and perceived workload was corroborated with existing literature (Adams & Bond, 2000; Kalisch et al., 2011; Lindqvist et al., 2014; Tao et al., 2018). As a matter of fact, higher workload demands would result in excess physical and mental work required by the individual nurse, as well as temporal stress and anxiety, resulting in a lower satisfaction rate (Hayes et al., 2015; Wang et al., 2012). Hence, engagement of nurse managers and healthcare leaders in strategies that lower the workload demands for nurses such as efficient scheduling and more collaboration between the nurses and other healthcare staff can eventually improve the nurses’ shift‐work satisfaction and ultimately improve patient care outcomes.

Our findings also showed that a higher number of rationed tasks per day shift was associated with shift‐work poor satisfaction among participating RNs. Specifically, poorer nurses’ shift‐work satisfaction during the day shift was associated with rationing of partial and sponge bath care, setting patient care plans, documenting and evaluating care, emotional and psychological support, positioning patients, preparing patients for tests and therapies, supporting patients with food and oral intake, and monitoring of patients (Appendix: Table A1). Given that shift‐work satisfaction is a self‐reported measure, the individual RN may experience a demoralized sensation reflected in being unsatisfied with the quality and quantity of work performed on that specific shift with increased instances of postponing or not performing a task that should have been otherwise performed. For example, in a study conducted in the United Kingdom, nurses were five times more probably to have a demoralized sensation when reporting rationing of care (Senek et al., 2020). This phenomenon is referred to as “self‐punishment” during which nurses tend to punish themselves for not being satisfied about their work, as an emotional regulation strategy and an opportunity to reflect and learn from the rationing of care transgression (de Vel‐Palumbo et al., 2018). Conforming to our findings, Kalisch et al. (2011) and White et al. (2019) found that nurses who reported higher satisfaction with their job are less probably to report missed care, particularly related to psychological support for patients, adequate surveillance, and care planning on their respective patient care units (Kalisch et al., 2011; White et al., 2019). Therefore, employed strategies in healthcare organization including better scheduling approach and higher level of teamwork and cooperation could lower nurses’ perceived workloads and rationing of nursing care, and eventually improve shift‐work satisfaction among nurses.

## 7 STRENGTHS AND LIMITATIONS

To the best of our knowledge, this is the first longitudinal study on nurses’ shift‐work satisfaction estimating the association over time and through repeated assessments between shift‐work satisfaction and implicit rationing of nursing care and key work environment factors, including workload, patient‐to‐nurse ratio, and the type of working shift and working unit.

Despite the novelty of the present study, it is important to highlight several limitations. First, data were gathered from only one hospital and using a convenience sampling approach, which may not characterize associations in other local or regional hospitals, limiting the generalizability of our findings. Nevertheless, all the 90 recruited RNs answered our longitudinal survey which provides a comprehensive data in that setting. Second, response and reporting bias are common phenomena in self‐reported healthcare research (Rosenman et al., 2011). The reasons for individuals giving biased ratings of self‐assessed behaviour can range from misunderstanding of some survey questions to “social‐desirability” bias, even in anonymous surveys (Rosenman et al., 2011). However, we addressed social‐desirability bias by ensuring complete anonymity and low levels of coercion through instructing the participating RNs to fill the survey alone without interference from other RNs working on the same shift. We also emphasized that each RN place the filled survey in a sealed envelope which can only be opened by a member of the research team at the end of the data collection phase. Third, in the data analysis phase, we could not adjust for the socio‐demographic characteristics that were collected in an aggregated manner only, as recommended by the ethical committee to protect the individual nurse anonymity. Forth, unit‐level work environmental elements including nurse manager leadership, patient safety and teamwork climate were missing in the longitudinal questionnaire to avoid the time burden of repeated measuring points and sustain high response rate. Finally, our analysis was based on observing nurses’ shift‐work satisfaction through a linear time trend in the GLMM models. Lagged time effects were not accounted for. Based on our study design, we asked nurses about their shift‐work satisfaction in their respective working shift, which was analysed in relation to the rationing of care, workload and staff level demands on that specific shift.

## 8 CONCLUSION

This longitudinal observational study revealed statistically significant variability of nurses’ shift‐work satisfaction between the individual nurse responses, yet no variability was noted in the responses of nurses working in the medical unit versus the surgical unit. Self‐reported shift‐specific workloads and rationing of nursing care acted as strong predictors for the shift‐work satisfaction among RNs providing acute care. In contrast, the unit‐shift‐specific patient‐to‐nurse ratio was not associated with the nurses’ shift‐work satisfaction. This shows the importance of perceived shift‐specific workloads and rationed care in predicting the nurses’ shift‐work satisfaction. Furthermore, our findings encourage nurse managers and healthcare leaders to implement shift‐oriented strategies that monitor and lower the workload demands for nurses. Remedial interventions may include optimization of nurse staff scheduling and establishment of cooperative and teamwork environment while observing rationing of care. These strategies promote nurses’ satisfaction about the work they performed on their respective shifts and alleviate the observed deficiency of nurses in the Lebanese healthcare sector. Nevertheless, it should be noted that the objective workload assessment in terms of patient‐to‐nurse‐ratio was not a statistically significant predictor for the nurses’ shift‐work satisfaction as its variation across time was not statistically significant, and hence the mechanism of patient‐to‐nurse staffing should be further investigated. For future research, we also recommend assessing the effect of organizational workplace factors other than workload including the nurse managers’ leadership and teamwork establishments.

## CONFLICT OF INTEREST

The authors have declared no conflict of interest.

## AUTHOR CONTRIBUTIONS

MA, ME, SD, MS and DA made substantial contributions to conceptualization and design, methodology, data curation, data preparation, data formal analysis, visualization, interpretation, investigation and validation. MA, ME and SD involved in supervision and project administration. MA and ME made substantial contributions to software. MA, ME and SD involved in writing (original drafting) of the manuscript. MA, ME, SD, MS, DA and HA involved in funding acquisition. MA, ME, SD, MS, DA and HA given final approval of the version to be published. Each author should have participated sufficiently in the work to take public responsibility for appropriate portions of the content. MA, ME, SD, MS, DA and HA agreed to be accountable for all aspects of the work in ensuring that questions related to the accuracy or integrity of any part of the work are appropriately investigated and resolved. MA, ME, SD, MS, DA, and HA involved in writing (Review and editing) of the manuscript.

## Data Availability

The data that support the findings of this study are available on request from the authors. The data are not publicly available due to privacy or ethical restrictions.

## 1

**TABLE A1:** Generalized linear mixed models showing the association between nurses’ shift‐work satisfaction and each of the 14 rationed nursing tasks in the day shift (*n* = 1,032 responses, *n* = 64 RNs) and in the night shift (*n* = 271, *n* = 34 RNs)

Nurse satisfaction in the day shift OR (95% CI)
Nurse satisfaction in the night shift OR (95% CI)
Rationing of providing daily living support		
Partial/Sponge bath	0.47 (0.24, 0.93)*	1.90 (0.35, 10.45)
Skin care	0.60 (0.36, 1.01)	3.42 (0.85, 13.77)
Oral care	0.68 (0.41, 1.14)	1.84 (0.52, 6.50)
Rationing of documentation		
Patient care plans	0.44 (0.27, 0.74)**	0.96 (0.91, 1.03)
Documenting/evaluating care	0.27 (0.14, 0.53)**	0.72 (0.16, 3.18)
Rationing of rehabilitation, instruction and education		
Emotional/Psychological support	0.26 (0.15, 0.46)**	0.79 (0.25, 2.47)
Training/educating patients	0.57 (0.31, 1.03)	1.59 (0.48, 5.30)
Rationing of monitoring and Safety		
Positioning patients	0.42 (0.23, 0.78)**	2.35 (0.45, 12.17)
Rationing of caring and support		
Preparing patients for tests/therapies	0.39 (0.17, 0.93)*	1.34 (0.37, 4.79)
Assessment for newly admitted patients	0.55 (0.26, 1.18)	0.94 (0.23, 3.87)
Supporting patients with food/oral intake	0.46 (0.25, 0.86)*	1.67 (0.48, 5.81)
Preparing patients for hospital discharge	1.21 (0.61, 2.37)	1.24 (0.36, 4.29)
Attending to patients who rung promptly in <5 min	0.85 (0.49, 1.49)	1.79 (0.41, 7.91)
Monitoring patients	0.48 (0.27, 0.86)*	0.99 (0.20, 4.77)

Note

All models are adjusted for the time trend (day shifts: T1–T61; night shifts: T62–T91), weekend/holiday (0 = weekday, 1 = weekend/holiday), workload (unweighted mean score of mental, physical, temporal, frustration, effort, and performance scale items), working unit (1 = medical unit, 2 = surgical unit) and patient‐to‐nurse ratio (number of patients÷number of registered nurses per unit per shift).

* *p*‐value < .05,

** *p*‐value < .01.
